# Dual-Tower Counterfactual Session-Aware Recommender System

**DOI:** 10.3390/e26060516

**Published:** 2024-06-14

**Authors:** Wenzhuo Song, Xiaoyu Xing

**Affiliations:** 1 College of Information Science and Technology, Northeast Normal University, Changchun 130117, China; xingxiaoyu@nenu.edu.cn; 2 Key Laboratory of Symbolic Computation and Knowledge Engineering of Ministry of Education, Jilin University, Changchun 130012, China

**Keywords:** information system, neural networks, session-aware recommender system, personalized session-based recommendation, machine learning

## Abstract

In the complex dynamics of modern information systems such as e-commerce and streaming services, managing uncertainty and leveraging information theory are crucial in enhancing session-aware recommender systems (SARSs). This paper presents an innovative approach to SARSs that combines static long-term and dynamic short-term preferences within a counterfactual causal framework. Our method addresses the shortcomings of current prediction models that tend to capture spurious correlations, leading to biased recommendations. By incorporating a counterfactual viewpoint, we aim to elucidate the causal influences of static long-term preferences on next-item selections and enhance the overall robustness of predictive models. We introduce a dual-tower architecture with a novel data augmentation process and a self-supervised training strategy, tailored to tackle inherent biases and unreliable correlations. Extensive experiments demonstrate the effectiveness of our approach, outperforming existing benchmarks and paving the way for more accurate and reliable session-based recommendations.

## 1. Introduction

As e-commerce and content streaming services grow increasingly sophisticated, the pursuit of personalized user experiences has propelled the advancement of information theory applications within these domains. This has given rise to the burgeoning field of next-item prediction in session-based recommendation systems (SBRSs), where concepts drawn from entropy and information-theoretic frameworks are now being used to augment personalization strategies [1,2]. Standard session-based recommendations leverage current user interactions within sessions to predict next items. However, next-item prediction has evolved into a more comprehensive approach with the evolution of session-aware recommendation systems (SARSs) [3], which integrate the understanding of users’ static long-term preferences alongside dynamic short-term preferences within a session. This evolution has sparked a growing scholarly interest since it poses additional complexities in unraveling how long-term and short-term preferences collectively shape the user’s choices for the next item within a session [4].

Despite the popularity of this research area, current methodologies predominantly employ machine learning models focused on recognizing patterns of correlations present in the data [5]. Such a practice often culminates in models that inadvertently learn spurious correlations and are susceptible to biases like selection bias, leading to suboptimal performance [6]. A notable shortfall in these works is the underrepresentation of the causal relationships between preferences and user choices, necessitating a paradigm shift in how recommendations are approached. Moreover, extant studies minimally explore the exact role of static long-term preferences within session-aware recommendations. Typically, these preferences are merely considered an external data source from which deep learning techniques extract potentially useful features, without a causal perspective on their effect and significance [1,2].

Prompted by these challenges, our work is motivated by the need to reconceptualize session-aware recommendations by examining the causal relationships between static long-term and dynamic short-term preferences and their resultant impact on the next-item choice from a causal viewpoint. By adopting a causal lens, we aim to strengthen the robustness of predictive models against unreliable correlations, thereby enhancing performance. Furthermore, our work delves into the practical role that static long-term preferences play by addressing counterfactual questions such as “how likely is the static long-term preference to make a difference on the next-item selection in a session”.

In this paper, we introduce a novel perspective by dissecting and analyzing the effects of static long-term preferences on next-item prediction tasks in session-aware recommendations from a counterfactual causal framework [7]. This framework paves the way for a deeper understanding of the intricate dynamics at play and the development of a counterfactual learning model tailored for this task. The model is designed to mitigate the learning of spurious correlations and data biases, thereby providing a more reliable and accurate recommendation system. Additionally, from a technical standpoint, we propose a dual-tower architecture alongside a counterfactual-enhanced data augmentation approach to refine the training process inspired by the recent works in counterfactual learning [8], aiming to develop an unbiased predictor for counterfactual questions using two decoder models for factual and counterfactual data. This approach is inspired by the 2016 ICML work of Johansson et al. [9] that utilizes domain adaptation for outcome prediction (i.e., next-item prediction in our work) across different treatment conditions (i.e., static long-term preferences of factual and counterfactual sessions in our work), encouraging similar representations of contextual variables (i.e., similar dynamic short-term preferences in our work). This representation-based counterfactual learning methodology [8,9] has successfully been applied to graph learning, causal learning, and recommender systems. This study further incorporates a novel self-supervised learning loss to augment the learning mechanism instead of the additional balance loss in [8,9] to align the representations of dynamic short-term preferences [5]. In essence, our primary contributions are as follows.

Contribution 1: We introduce a counterfactual causal framework specifically tailored for session-aware recommender systems. This innovative framework is designed to decipher the complex influence of static long-term preferences and is a step towards understanding user behavior in session-aware scenarios more profoundly. Unlike traditional machine learning/deep learning models, which aim to fit associative patterns in the data, the DTC model leverages causal learning and counterfactual learning to capture robust causal relationships in predicting users’ next-item choices. Our approach minimizes the risk of the model learning biased associations.

Contribution 2: At a technical level, we have conceptualized a novel dual-tower architecture that adeptly balances factual sessions and counterfactual sessions, which, when combined with our counterfactual data augmentation procedure, effectively mitigates the inherent biases often present in typical recommender systems. Our counterfactual data augmentation is pioneering in this domain, creating factual–counterfactual sessions that embed significant causal relationships. The dual-tower structure allows for simultaneous prediction of factual and counterfactual sessions, incorporating multi-task learning to identify causal relationships in the augmented data. We also expand the self-supervised loss methodology to encapsulate counterfactuals, leveraging repeated user interactions with items in sessions as a signal for dynamic short-term preference.

Contribution 3: The efficacy of our proposed methodologies has been substantiated through extensive experimentation, wherein our model has exhibited superior performance over several benchmarks and existing state-of-the-art systems. These experiments not only reinforce the validity of our contributions but also provide a robust framework for future explorations into refining recommendation strategies. Specifically, in Section 4.2, we compare the DTC model with state-of-the-art and representative models, affirming superior performance. Section 4.3 provides an ablation study to decipher the contributions of the counterfactual framework, data augmentation, dual-tower architecture, and self-supervised learning. We further discuss our model’s limitations in addressing dynamic short-term preferences in Section 4.4.

## 2. Related Work

### 2.1. Session-Aware Recommender Systems

Session-aware recommender systems have emerged as an advanced variant of the traditional session-based recommender systems, augmenting the predictive prowess by assimilating users’ long-term preferences with their spontaneous inclinations within active sessions [3]. This deliberate integration attempts to bridge the gap in the predictive narrative that SBRSs leave untold by solely relying on intra-session interactions [10].

The standard SBRSs are designed to forecast the users’ next interest item based on active session data. Initial methods like Markov Chain-based models and Recurrent Neural Network (RNN)-based models have predominantly focused on modeling the items’ sequential patterns within a session [11,12,13,14,15]. However, these models operate under a strong sequential dependency assumption, which often does not reflect the multifaceted nature of user–item interactions. This incongruence calls into question the effectiveness of RNNs in environments where the order of interactions may not be strictly linear or highly correlated.

In pursuit of refining SBRSs, latent-factor-based models are proposed for learning better representations [16,17,18,19]. In recent years, attention-based models have been introduced to ascertain the relevance of each item within the session context for more accurate next-item predictions [4,20,21]. Through this mechanism, models assign varying weights to items, targeting those with a higher propensity to influence the user’s subsequent choice. Even so, such systems exhibit a propensity to favor popular items, which leads to a biased recommendation landscape that marginalizes less prevalent items.

Recently, graph neural networks (GNNs) have been widely adopted in SBRSs to decipher the intricate item transitions within sessions [22,23]. GNNs stand out by effectively capturing the complex item transition patterns, surpassing the capabilities of traditional sequence-based approaches. Despite their ingenuity, the standard GNN applications in SBRSs continue to overlook critical user-specific information, like long-term preferences, not readily deducible from session interactions.

Contrastingly, session-aware systems like Hierarchical Recurrent Neural Network (HRNN) models and II-RNN squarely address this by encoding user sessions and items separately before unifying their representations for item prediction [24,25]. Other models delve into historical interactions to extract a user’s immutable long-term preference, contributing to a more holistic recommendation system.

However, our work stands distinct in aiming to decipher the causality behind the interplay of static long-term preferences, dynamic short-term preferences, and their joint influence on next-item selection within SARSs. The proposed model within this paper employs counterfactual learning to methodically unravel these causal relationships.

### 2.2. Counterfactual Learning for Recommender Systems

Counterfactual learning for recommender systems has been pivotal in tackling a slew of inherent biases and shortcomings within the recommendation process [26,27]. Debiasing efforts like the MACR (model-agnostic counterfactual reasoning) framework specifically target the ubiquity of popularity bias in recommender systems [28]. Similarly, initiatives like the CR model address the distortion sustained from clickbait bias, providing recommendations that transcend misleading engagement metrics [29].

Furthermore, the sparsity of user–item interactions has been addressed by papers proposing novel data augmentation techniques to enhance sequential recommendation quality [30,31,32,33]. Counterfactual reasoning has also been leveraged to provide explainability in recommendations, as well as to promote fairness based on causal notions, essentially advocating for a more equitable recommendation system [34,35].

Our work aligns with a recent model named COCO, where counterfactual learning methods are employed to account for out-session causes such as promotional events [5]. Unique to our work is the approach that uses counterfactual learning methodologies to scrutinize the role of static long-term preferences in the next-item prediction task within SARS. Additionally, COCO is grounded in the hypothesis: “If an item (i.e., the next item) appears in the context of the target session, the user is more likely to select the item due to the ISCs (i.e., the dynamic short-term preference of the user in this session)” [5]. Our current study extends this hypothesis to encompass counterfactual sessions. Specifically, under the condition of similar dynamic short-term preferences ensured by the Counterfactual Interactions Matching method, an item appearing in either the factual or counterfactual session implies a higher likelihood of selection by the user. This sheds new light on a relatively underexplored facet of recommendation systems and underscores the need to discern the subtle mechanisms shaping user preferences and their decision-making processes in e-commerce platforms and beyond.

## 3. Method

### 3.1. Problem Statement

In our research, we articulate the set of users with the notation $U=\{u_{1},\;\ldots ,\;u_{\left|U\right|}\}$ and the set of items by $V=\{v_{1},\;\ldots ,\;v_{\left|V\right|}\}$ within a dataset. For each user u∈U, they have engaged with a sequence of user–item interactions, which are chronicled as a list delineating the items involved in these interactions. When delving into the domain of session-aware recommender systems (SARSs), these user interactions are organized into a sequence of sessions. Each session encapsulates a list detailing a user’s interactions nested within a compact timeframe. We designate a session using the symbol $s=\{v_{1}^{s},\;\ldots ,\;v_{t}^{s}\}$, where the subscript assigned to each item within *s* signifies its sequence of occurrence during the session. The primary objective of a SARS involves forecasting the subsequent interaction in a focal session *s* concretely, predicting the next item $v_{t+1}^{s}$. This prediction is grounded on accessible knowledge and information such as the user’s preferences and the contextual dynamics of the session.

In the realm of counterfactual learning, attention is centered upon three pivotal variables: context, treatment, and outcome [8]. That is, provided the context of a data point, our interest lies in the alteration of the outcome when a treatment shifts from an observed reality (often referred to as the ‘factual’) to an unobserved alternative (known as the ‘counterfactual’), which in turn informs us about the change in the outcome (known as the individual causal effect). Within the scenario of our study, considering a session (i.e., a data point), we perceive the observed session *s* as the context—a reflection of *u*’s dynamic short-term preference. The treatment is conceived as the user *u*’s static long-term preference, and the outcome is embodied by the next item $v_{t+1}^{s}$ of the session *s*.

### 3.2. Overview of the Proposed Framework

This section introduces our novel counterfactual session-aware recommender system model, which we refer to as the Dual-Tower Counterfactual Session-based Recommender System, abbreviated as DTC-SBRS or simply DTC. The DTC model is designed to harness a causal model to construct more robust causal relationships within the data. Its objective is to mitigate the model’s acquisition of unreliable correlational information, thereby enhancing the performance of the recommendation system. The core idea of the causal model is to guide the learning of causal relationships within the dataset by addressing the counterfactual question: “How likely does the static long-term preference influence the selection of the next item in a session?” The DTC model comprises several key steps:

**(1)**  **Counterfactual Interactions Matching**: Initially, for each session in the training set, we generate a corresponding counterfactual session utilizing the nearest neighbor matching to enhance the training data.
**(2)**  **Self-supervised-based Dual-Tower Counterfactual Learning**: This stage employs the idea of data augmentation to train the model, which involves using the original sessions (known as factual sessions) from the training set along with counterfactual sessions generated through the Counterfactual Interactions Matching step. The recommendation model being trained utilizes an attention-based architecture and features a dual-tower structure. This dual-tower approach encourages parallel learning of both factual and counterfactual sessions, promoting the model to discern causal relationships by contrasting the different static long-term preferences between factual and counterfactual sessions. Specifically, one tower targets the prediction of the next item in the factual session, while the other tower focuses on the next item in the counterfactual session. Multi-task learning strategies are applied to assimilate the counterfactual insights from both factual and counterfactual sessions. To further motivate the model to learn the different static long-term preferences and their causal effects on next-item selection, our paper proposes a novel training objective function based on self-supervised learning. The main concept is to control dynamic short-term preferences and the causal relationship between these preferences and the next-item decision. Under these conditions, distinct static long-term preferences of factual and counterfactual sessions lead to varied choices of the next item.
**(3)**  **Intervention-based Counterfactual Recommendation**: The DTC model integrates ideas from collaborative filtering and counterfactual inference to predict the next item in a target session. Conventionally, collaborative filtering techniques would first identify a small quantity of neighbor sessions, or similar sessions, within the dataset that are akin to the target session. The items from these neighbor sessions are then predicted as the potential next items for the target session. The challenge with this approach, however, lies in its heavy reliance on the computation method for session similarity. Since SARSs must consider finding similar sessions with comparable dynamic short-term and static long-term preferences, the task becomes challenging, often resulting in the retrieval of noisy data or similar sessions laden with disproportionately popular items. Building on this, our research introduces an Intervention-based Counterfactual Recommendation method founded on the principles of counterfactual inference. Specifically, for a similar session $s^{\prime }$ derived from the dataset for the target session *s*, we employ counterfactual inference to hypothesize: what would be the next item for $s^{\prime }$ if the static long-term preference was the same as that of the user corresponding to *s*. This counterfactual inference-based next-item prediction is anticipated to closely align with the actual next item in the target session *s*, compared to direct predictions based on $s^{\prime }$ and its associated user’s long-term preference.

The forthcoming subsections will delve into the specifics of each part of the DTC model.

### 3.3. Counterfactual Interactions Matching

The aim of ‘Counterfactual Interactions Matching’ is to generate augmented data to train the model proposed in this paper. From a causal perspective, our core idea at this stage is to acquire a pair of data points that share the same context variable (in this case, the dynamic short-term preference reflected in the session) but differ in their treatment (in this case, the static long-term preference associated with the user of the session) and the outcome (which is the next item in the session). This is arranged so that by comparing the variation in outcome under different treatment conditions, we can understand the causal relationships within the data.

Specifically, for each session *s* in the training set (referred to as the factual session), with an associated user *u*, we search the training set for one of the most similar sessions from a different user $u^{\prime }$, denoted as session $s^{\prime }$ (referred to as the counterfactual session). The formula for calculating the similarity between sessions is as follows:

(1) $$sim\left(s,\;s^{\prime }\right)=\frac{|s\;\cap \;s^{\prime }|}{|s\;\cup \;s^{\prime }|}$$

We then obtain $s^{\prime }$ as follows:

(2) $$s^{\prime }=\underset{s^{\prime }}{\mathrm{argmax}}\;sim\left(s,\;s^{\prime }\right)$$

Since $s^{\prime }$ originates from another user $u^{\prime }$, the treatment variable for $s^{\prime }$ differs from that of *s* (meaning *s* and $s^{\prime }$ have different static long-term preferences). However, since *s* and $s^{\prime }$ are similar, their dynamic short-term preferences are nearly identical. Under this design, *s* and $s^{\prime }$ serve as a pair of sessions with similar context (dynamic short-term preference) and outcomes (the next items in the session) but different treatments (static long-term preference).

By applying the method described above to every session *s* in the training set to compute a corresponding counterfactual session $s^{\prime }$, we merge all factual sessions from the training set with the generated counterfactual sessions to obtain an enhanced training dataset.

### 3.4. Self-Supervised-Based Dual-Tower Counterfactual Learning

**Figure 1:**
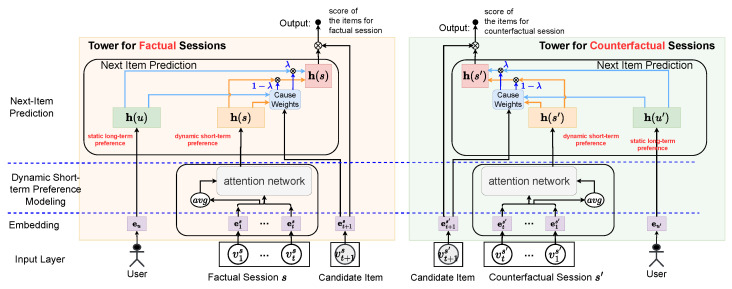
The dual-tower structure of the proposed DTC model.

The goal of this section is to train a recommendation system model that can learn the causal relationships within the model. Specifically, we propose two approaches to achieve this objective:

The first approach is for the model to learn the causal relationships between static long-term preferences, dynamic short-term preferences, and next items by comparing the factual and counterfactual sessions in the augmented dataset. To this end, we designed a neural network model with a dual-tower structure, where each tower is dedicated to learning the data distribution of either factual or counterfactual sessions. Both towers have identical structures and share parameters, implemented using attention-based neural networks. The dual-tower framework is depicted in Figure 1.

**Modeling User Preferences**: We use a user’s embedding to represent static long-term preference, denoted as $h\left(u\right)=e_{u}\in R^{d}$. The calculation for dynamic short-term preference h(s) is as follows:

(3) h(s)=attention(avg(E(s), E(s), E(s)))

Here, $E\left(s\right)\in R^{|s|\times d}$ is the matrix of embeddings corresponding to all interactions within *s*, and avg is a function to calculate the average of vectors. The attention neural network attention(q, K, V) is defined as follows:

(4) $$attention\left(q,\;K,\;V\right)=\sum_{i}\alpha \left(q,\;K_{i}\right)\times V_{i},$$

with $q\in R^{d}$ and $K,\;V\in R^{d\times l}$ representing matrices made of *l* vectors. Attention scores λ are computed as follows:

(5) $$\alpha \left(q,\;K_{i}\right)=softmax_{i}\left(K_{i}\cdot q^{T}/\sqrt{d}\right).$$

**Next-Item Prediction**: Given a pair of a factual session *s* and its corresponding counterfactual session $s^{\prime }$, we predict the next item for both *s* and $s^{\prime }$:

(6) $$\begin{array}{cc} \; & p\left(v|s,\;u\right)=\delta \left(EH_{s,u}^{T}\right)\\ \; & p\left(v^{\prime }|s^{\prime },\;u^{\prime }\right)=\delta \left(EH_{s^{\prime },u^{\prime }}^{T}\right) \end{array}$$

Here, *v* and $v^{\prime }$ represent the variables for the next item of *s* and $s^{\prime }$, respectively, $E\in R^{|V|\times d}$ is the matrix of embeddings for all items, and the softmax function $\delta {\left(x\right)}_{i}$ is defined as follows:

(7) $$\delta {\left(x\right)}_{i}=\frac{e^{x_{i}}}{\sum_{j=1}^{\left|x\right|}e^{x_{j}}}.$$

Since the model needs to predict the next item for both factual and counterfactual sessions, and the model used for prediction is symmetrical in structure and parameters, we describe the model as having a dual-tower structure. *H* in the above equations is computed as follows:

(8) $$H_{s,u}=\lambda h\left(s\right)+\left(1-\lambda \right)h\left(u\right),$$

where

$$\lambda \left(h\left(s\right),\;h\left(u\right)\right)=1/\left(1+e^{-W^{T}\left(h\left(s\right)\|h\left(u\right)\right\|e_{v})+b}\right)$$

This represents the weight of dynamic short-term preference, with 1−λ as the weight for static long-term preference. $W\in R^{d}$ and b∈R are learnable parameters, ‖ denotes the vector concatenate operation, and $e_{v}$ signifies the embedding of item *v*.

**Training Dual-Tower Model with Cross Entropy Loss**: Next-item prediction can be viewed as a multi-class classification problem predicting the probability of all items being the next item. For both factual and counterfactual sessions, we use Cross Entropy Loss as the objective function for the two prediction targets of the dual-tower:

(9) $$\begin{array}{cc} L_{1}=-\frac{1}{\left|S\right|}\sum_{s\in S}\{[ & \log p(v=v_{+}^{s}|s)\\ \; & +\sum_{v_{-}^{s}\in V,v_{-}^{s}\neq v_{+}^{s}}\log\left(1-p\left(v=v_{-}^{s}|s\right)\right)]\\ \; & +\epsilon [\log p(v=v_{+}^{s^{\prime }}|s^{\prime })\\ \; & +\sum_{v_{-}^{s^{\prime }}\in V,v_{-}^{s^{\prime }}\neq v_{+}^{s^{\prime }}}\log\left(1-p\left(v=v_{-}^{s^{\prime }}|s^{\prime }\right)\right)]\}, \end{array}$$

where *S* is the set of all factual sessions in the training set, $v_{+}^{s}$ and $v_{+}^{s^{\prime }}$ are the real next-items of *s* and $s^{\prime }$, respectively, $v_{-}^{s}$ and $v_{-}^{s^{\prime }}$ are next-items obtained through random negative sampling, and ϵ is the weight of prediction for counterfactual session.

**Self-supervised Learning for the DTC Model**: By training the above-mentioned dual-tower model using both factual and counterfactual sessions, we can analyze next-item selections under contrasting static long-term preferences, keeping the dynamic short-term preference constant. However, this overlooks another significant factor influencing users’ next-item selections, namely how users decide on next-items based on dynamic short-term and static long-term preferences. For example, in certain session scenarios, users might only consider static long-term preferences, leading to unrelated items within the same session. Thus, the second approach to enhancing the model’s performance is to propose a loss function based on self-supervised learning to assist the model in learning the causal relationships included among the dynamic short-term preference, static long-term preference, and next-item variables.

Specifically, we introduce a self-supervised loss to aid the model in learning the attention weights measuring the importance of dynamic short-term preference and static long-term preference in the dual-tower model, namely the λ values from the equations above. Our approach is inspired by the hypothesis presented in the WWW 2023 study [5], which asserts that “If an item (i.e., the next item) appears in the context of the target session, the user is more likely to select the item due to the inner-session causes (i.e., dynamic short-term preferences within this session).” Our research broadens this hypothesis to include counterfactual sessions. Under the premise of matching dynamic short-term preferences facilitated by the Counterfactual Interactions Matching method, we assume that when the real next-item is present in either the factual or counterfactual session, the weight of dynamic short-term preference should be greater, and conversely, the weight of static short-term preference should be smaller. This hypothesis draws from real-world observations across various domains such as music recommendations and online shopping. In these fields, users frequently engage with specific items over time, like repeatedly listening to a favored song [36]. Such repetitive interactions highlight a user’s preference over a given period, exemplifying a dynamic short-term preference that can be effectively modeled to enhance recommendation accuracy. Consequently, we posit that the presence of the actual next item in either the factual or counterfactual session should result in a higher weight for dynamic short-term preference, and conversely, the weight of dynamic short-term preference should be smaller. Based on this, we define the following pseudo-label to guide the training of λ within the DTC model:

$$y\left(v,\;s\right)=1\left[v\in s\;\mathrm{or}\;v\in s^{\prime }\right],$$

where 1[condition] is defined as follows:

$$1\left[condition\right]=\left\{\begin{array}{cc} 1, & \mathrm{if}\;condition\;\mathrm{is}\;\mathrm{True},\\ 0, & \mathrm{if}\;condition\;\mathrm{is}\;\mathrm{False}. \end{array}\right.$$

Based on this assumption, we calculate the self-supervised loss as follows:

(10) $$L_{2}=\frac{1}{\left|S\right|}\sum_{s\in S}BCE\left(\lambda ,\;y\left(v_{+}^{s},\;s\right)\right)+BCE\left(\left(1-\lambda \right),\;y\left(v_{+}^{s},\;s\right)\right)$$

where BCE is the Binary Cross Entropy, defined as follows:

BCE(x, y)=y·logx+(1−y)·log(1−x)

Finally, we combine $L_{1}$, the loss for training the model to predict the next-item from the augmented data, with $L_{2}$, the loss helping the model to learn the causal relationships among users’ dynamic short-term preference, static long-term preference, and next-item variables, to form the DTC model’s loss function:

$$L=L_{1}+\beta *L_{2},$$

where β∈[0, 1] is a trade-off parameter.

**Discussion**: The dual-tower structure conceptually originates from the work presented at ICML 2022 [8], aiming to develop an unbiased predictor for counterfactual questions using two decoder models for factual and counterfactual data. This approach is inspired by the 2016 ICML work [9] that utilizes domain adaptation for outcome prediction (i.e., next-item prediction in our work) across different treatment conditions (i.e., static long-term preferences of factual and counterfactual sessions in our work), encouraging similar representations of contextual variables (i.e., similar dynamic short-term preferences in our work). This representation-based counterfactual learning methodology [8,9] has successfully been applied to graph learning, causal learning, and recommender systems. In our study, we opt for a self-supervised loss (Equation (10)) instead of an additional balance loss as in [8,9] to align the representations of dynamic short-term preferences.

### 3.5. Intervention-Based Counterfactual Recommendation

The objective of this section is to enhance the model’s performance in the prediction phase through counterfactual inference. In our work, we apply a collaborative filtering framework to predict the next item for a target session *s*, and we introduce counterfactual interventions for unreliable neighbor sessions found in the dataset by the collaborative filtering model.

Traditional collaborative filtering methods directly use the items from neighbor sessions as the next-item prediction for *s*. However, this approach often yields neighbor sessions that are not similar to *s*, primarily because traditional methods compute session similarity by considering only the interactions within *s*, neglecting the long-term preferences of the user associated with *s*. In this paper, we propose a method based on counterfactual inference to calculate neighbor sessions that are similar to the current session. The neighbor sessions obtained by this method are generated by the model rather than those observed by traditional methods. Therefore, we can control the generation process of neighbor sessions to produce sessions that are similar to the target session while also considering diversity, thus improving the performance of collaborative filtering.

Specifically, given a target session *s*, we first identify the *K* most similar neighbor sessions $s_{i}^{\prime }\in S$, i∈[1, K] from the training set by calculating session similarity, along with their corresponding users $u_{i}^{\prime }\in N_{s}\left(u\right)$, where $N_{s}\left(u\right)$ represents the set of users from *s*’s neighbor sessions. Next, we use the DTC model introduced in the previous section to answer the counterfactual question, “How would user $u^{\prime }$ select the next item in session *s*?” by calculating the following formula:

$$p(v|s,\;u^{\prime })$$

Subsequently, we predict the next item for *s* using the following formula:

$$p\left(v|s,\;u\right)=\frac{1}{C}\sum_{u_{i}^{\prime }\in N_{s}\left(u\right)}sim\left(s,\;s_{i}^{\prime }\right)\times p\left(v|s,\;u_{i}^{\prime }\right),$$

where *C* is the normalization factor.

This method leverages the precision of individual user preferences and the power of collaborative filtering to yield a more refined, counterfactually informed prediction for the next item in the target session.

## 4. Experiments

### 4.1. Experimental Settings

**Baseline Algorithms:** Our comprehensive comparison includes a spectrum of pioneering session-based and sequential recommendation systems, each underpinned by distinct computational paradigms including collaborative filtering, state-of-the-art recurrent networks, attentional, memory-augmented, and graph-based neural networks. Specifically, our ambit entails five session-based recommendation systems (SBRSs)—SKNN, GRU4Rec, STAMP, CSRM, and SRGNN—paired with six session-aware recommendation systems (SARSs) that encompass both enduring long-term user preferences and the dynamic nature of short-term preferences. These systems comprise HGRU, II-RNN, SASRec, BERT4Rec, INSERT, and COCO, each contributing a unique approach for gleaning insights from user interaction data.

- **SKNN**: This straightforward model introduces a collaborative angle to the context of sessions, homing in on items from sessions that share similarities with the active session, much like comparing neighborhoods to find the best result [37].
- **GRU4Rec**: Unpacking the sequence of choices a user makes, GRU4Rec brings into use gated recurrent units within an RNN to capture the patterns of a user’s evolving interests during a session [13].
- **STAMP**: STAMP employs an innovative memory-based neural network enhanced with attention mechanisms to pinpoint precise user desires at a given moment, using this focus to guide the next-item recommendation [38].
- **CSRM**: A memory-neural-network-based SBRS that uses attention networks to learn session similarities and make recommendations based on neighbor sessions and their similarities [39].
- **SRGNN**: A graph-neural-network-based SBRS that represents sessions as graphs and employs a graph neural network to recommend items based on item transition patterns extracted from the graph [23].

The following five SARSs consider both static long-term preference and dynamic short-term preference:

- **HGRU**: HGRU employs a hierarchical RNN where one RNN models sequential patterns in the session context, and the other RNN learns a user’s preferences across their sessions [24].
- **II-RNN**: II-RNN utilizes information from the most recent session to complement and initialize the RNN modeling the target session [25].
- **SASRec**: SASRec is a self-attention-based sequential RS designed to model users’ interaction sequences. In this work, we concatenate all interactions of each user to form user sequences [40].
- **BERT4Rec**: BERT4Rec is a deep bidirectional self-attention-based sequential RS model. BERT4Rec adopts the Cloze objective and predicts an item based on its context and the user’s historical interactions [41].
- **INSERT**: INSERT is an SBRS that considers both users’ static long-term preferences and short-term preference in sessions. It is designed for next-item recommendations in short sessions based on few-shot learning and meta-learning [10].
- **COCO**: COCO is a state-of-the-art SARS and utilizes counterfactual learning to learn representations of inner-session causes and outer-session causes for better recommendation performance [5].

**Datasets:** We conduct experiments on two publicly available real-world datasets used in previous works:

- **Last.fm** (Last.fm and Delicious are from https://grouplens.org/datasets/hetrec-2011/, accessed on 1 May 2024.) used in [3] contains logs of users’ music listening behaviors in the Last.fm online music service.
- **Delicious** is a dataset that contains user tagging records in a social-network-based bookmarking system named Delicious.

**Data Preparation:** The process of preparing datasets mirrors the methodologies established in prior research on session-aware recommendation systems [3,5,10,25]. Our approach entails the following steps: initially, we filter out users and items exhibiting less than 10 interactions to remove noise and improve dataset quality. We then organize interactions into sessions based on a clear temporal criterion: if two consecutive interactions occurred within a 6 h timeframe, they are bundled into one session; if not, they are split across two distinct sessions. This is performed to capture meaningful interaction sequences. Sessions that do not provide adequate context for analysis—specifically, those with a single interaction or those with more than twenty—are also discarded. The refinement process aims to create sessions that are optimally sized for effective predictive modeling. Post-preprocessing, we summarize the core characteristics of the datasets as displayed in Table 1.

**Evaluation Protocol:** Our research adopted a robust 5-fold cross-validation method to ascertain consistently reliable results. The dataset’s session data were randomly segregated into five subsets of equal size. Each fold of the experiment used one of these subsets as the test set, while the remainder formed the training set. This rotation occurred five times to ensure comprehensive testing, and we calculated an average of the outcomes to present our findings. In an effort to eliminate any potential result bias, we standardized folds to include every user and each product in the training set. Furthermore, for the test sets, we randomly extracted half of the sessions to create the validation set. Within each testing session, we sequentially targeted each item, using preceding items as the session’s context for predictions. For every algorithm, we ranked items according to their recommendation scores and identified the top-K products. With these products, we calculated recall@K, NDCG@K, and MRR@K to quantify model effectiveness.

**Evaluation Metrics:** For performance metrics, we applied recall@K, normalized discounted cumulative gain (NDCG)@K, and Mean Reciprocal Rank (MRR)@K, established ranking measures used widely in the field for evaluating SBRS performance [5,40]. Recall@K measures the proportion of relevant items that are successfully retrieved within the top-K recommendations. It provides insight into the model’s ability to retrieve relevant items. NDCG@K considers the position of relevant items in the top-K recommendations, rewarding placements higher up in the list more heavily. It reflects both the relevance and rank of the recommended items. MRR@K focuses on the rank position of the first relevant item in the top-K recommendation list. Higher MRR@K values indicate that relevant items appear earlier in the recommendations.

**Fairness and Robustness:** To ensure the fairness of our evaluation, we employed a grid search method over hyperparameters for all models. We start by setting hyperparameters as recommended in the originating research to ascertain optimal performance for each algorithm. By systematically tuning each model, we aimed to minimize biases and provide a level playing field for comparison. We used an early stopping mechanism based on the validation set performance to prevent overfitting. This strategy not only enhances the generalization power of our models but also ensures that the reported performance metrics are reflective of real-world conditions. Our primary focus was the algorithms’ ability to recommend the next item in short sessions, an area where certain SARS models have shown particular strength.

**Reproducibility:** The key settings for the benchmark algorithms are as follows: KNN selects 500 similar sessions, CSRM utilizes 256 memory slots, II-RNN adopts 50-dimensional embeddings, INSERT considers 10 similar sessions, both SASRec and BERT4Rec implement 2 layers of attention with 2 heads each, and for COCO-SBRS, we apply early stopping contingent on R@20 from the validation set, limiting the process to a maximum of 50 epochs. We set the learning rate at 0.01, the session count at 10, and the trade-off parameter uniformly at 1 for all tests conducted. The implementation of the proposed DTC-SBRS leverages the PyTorch framework along with GPU acceleration to facilitate model training. We employ Xavier Initialization for the random generation of user and item embeddings, a prevalent technique in machine learning.

### 4.2. Recommendation Performance Evaluation

In the experiments conducted, we performed comparative evaluations to ascertain the efficacy of the newly developed Dual-Tower Counterfactua (DTC) against established session-aware recommender systems (SARSs). The aim was to determine the relative performance enhancements achieved through the implementation of DTC, as documented in our results reflected in Table 2.

Across two distinct datasets, we observed varying levels of performance among the competing algorithms. Notably, the initial subset comprising SKNN, GRU4Rec, STAMP, CSRM, and SR-GNN demonstrated subpar outcomes when placed in juxtaposition with their counterparts. This observation can be attributed to their primary focus on user short-term preference, neglecting static long-term preference. To illustrate, SKNN showed limitations in encapsulating complex item trends and individual user tastes in its similarity computation. STAMP, employing attention neural networks, excelled in distilling insights from sessions populated with less informative items. CSRM’s efficiency is ultimately dependent on its ability to discern session similarities using attention networks while conjointly harnessing a robust session encoding via memory neural networks. GRU4Rec and SR-GNN, each with unique mechanisms to capture sequential and transitional item patterns within sessions, find their performance tethered to the prevalence of these patterns within the dataset.

In contrast, algorithms like HGRU, II-RNN, SASRec, BERT4Rec, INSERT, and COCO extended their analytical purview, incorporating both dynamic short-term preferences and static long-term preference into their operational frameworks. HGRU adeptly adapts to changes in user preferences across consecutive sessions, utilizing a user-level RNN to portray this variability. II-RNN posits a stationary user preference model between sessions, leveraging the latest session data to enrich the contextual understanding of the targeted session. Both SASRec and BERT4Rec expand the interaction sequence to include the session of interest and all prior user interactions, thus harnessing rich data to extricate nuanced preferences utilizing advanced neural networks. INSERT, a leading algorithm for short-session item recommendations, employs a tailored session comparison module to capture insights across user interactions. According to Table 2, INSERT outshines its predecessors concerning most metrics. Nevertheless, it struggles to locate akin sessions within the dataset, especially when grappling with nuanced session-specific and external factors. COCO distinguishes itself by delivering marked advancements in both Recall and NDCG measures, which can be credited to its counterfactual computation model that effectively addresses session similarity challenges arising from sparse data, considering both session-intrinsic and extrinsic factors. Nonetheless, it fails to specifically target static long-term preferences and does not fully exploit counterfactual elements present within the dataset.

In the case of the proposed DTC model, it introduces an innovative approach by generating counterfactual sessions through counterfactual questions, subsequently augmenting the dataset. Leveraging this expanded dataset, the dual-tower structure coupled with multi-task learning empowers the model to discern the intricate causal relationships amongst long-term preferences, dynamic short-term interests, and the next-item predictions. Furthermore, the addition of self-supervised learning fosters a deeper understanding of the causal relationships interwoven between varying parameters.

### 4.3. Ablation Study

In this experiment, we examined the efficiency of different simplified versions of our DTC model, with the intent of analyzing the impact of each component in our counterfactual framework approach on the task of next-item recommendation.

We introduced three modified versions of the DTC model for our evaluation: (1) DTC w/o CF, which is essentially the principal model without the counterfactual framework, e.g., dual-tower structure, counterfactual data augmentation, and self-supervised learning; (2) DTC w/o dual-tower architecture, merely utilizing factual session data for training by setting the ϵ to zero; (3) DTC w/o SSL, essentially excluding the self-supervised loss as defined in Equation (10).

The comparative results, as provided in Table 3, show the performance metrics for the three simplified DTC models alongside the DTC model across the Delicious and Lastfm datasets. The results reveal that the DTC w/o CF lagged in performance, underscoring the significance of our proposed counterfactual framework. The results suggest that deep learning models, although considering both the static long-term and dynamic short-term user preferences, struggle to accurately model causal relationships within SARSs without the aid of counterfactual insights. It is evident from the worse performances of DTC w/o DT and DTC w/o SSL, when measured against the DTC model, that both the dual-tower structure and the application of self-supervised learning, including counterfactual data augmentation, serve as catalysts in amplifying the efficacy of our proposed DTC framework. Our dual-tower design enhances DTC’s output by engaging in an elaborate analysis of counterfactual learning and fostering the model’s ability to unravel complex causal relationships. Further, the integration of self-supervised learning encourages DTC with refined weights of causal relationships, supporting its capability to discern the true causal interplay amongst established long-term preferences, dynamic short-term interests, and next-item selections.

### 4.4. Recommendation Performance on Sessions with Different Lengths

**Figure 2:**
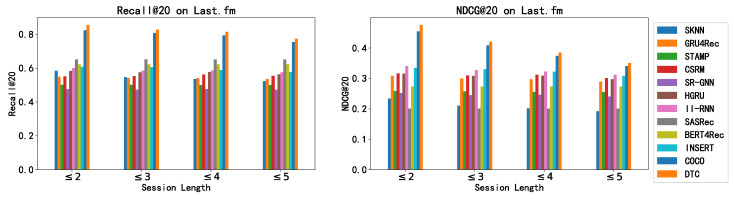
Recommendation performance of all methods on sessions with different lengths on Last.fm.

**Figure 3:**
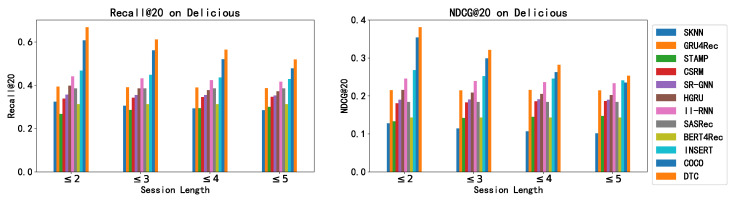
Recommendation performance of all methods on sessions with different lengths on Delicious.

In this group of experiments, we evaluate how well different methods perform on session data of varying lengths. Here, “session length” refers to the count of user interactions in a given session. With shorter sessions, there is naturally less behavioral data to extract information, decreasing the likelihood that item selections are influenced by dynamic short-term preferences that are based on recent interactions. The ability of each algorithm to perform across sessions of different lengths gives us insight into their capacity to grasp the relationship between static long-term preference, the dynamic short-term preference, and how these preferences play out in session-aware recommendation systems. We carried out these comparative tests on two distinct datasets, and the outcomes are presented in Figure 2 and Figure 3.

From the graphical data presented, we observe the following: (1) Across both datasets, our DTC model consistently surpasses the baseline approaches in terms of both Recall@20 and NDCG@20 metrics for sessions of length less than five. This confirms DTC’s robust approach to modeling the intricate cause-and-effect within SARS and its ability to sidestep spurious correlations that do not hold true causation. (2) As we analyze sessions with more interactions, a downward trend in DTC’s performance is apparent. The model shows its strengths clearly for sessions with five or fewer interactions. This decline suggests a potential weakness of DTC in harnessing valuable insights from lengthier sessions, potentially due to our choice to use a straightforward attention neural network to capture the dynamic short-term preference. Given these findings, we recommend that for datasets prone to longer sessions, an upgrade from the attention-based mechanism in the DTC to a more advanced session-encoding model would be advantageous for capturing the dynamic short-term preferences.

### 4.5. Hyper-Parameters Sensitivity Test

In this section, we conduct a set of tests to evaluate the response of the DTC model to changes in the hyper-parameter ϵ. This parameter is crucial as it governs the weights given to actual sessions versus generated counterfactual sessions in the training phase, as outlined in Equation (9).

**Figure 4:**
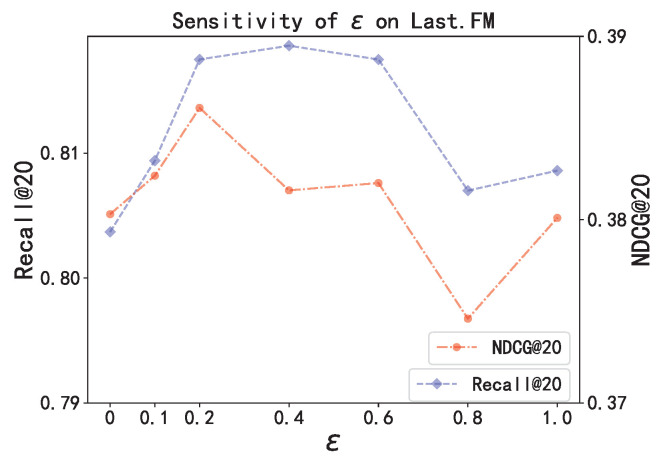
Sensitivity of ϵ on Last.FM.

The findings from these tests are graphically represented in Figure 4. Figure 4 illustrates that the DTC’s effectiveness improves when ϵ is increased up to 0.4, but it starts to drop off when ϵ exceeds this value. These observations suggest that the most effective balance between real and counterfactual session information for the DTC model is achieved when ϵ is set at 0.4.

## 5. Discussion

In this section, we discuss the characteristics, advantages, and limitations of the DTC model proposed in this work.

**Characteristics**: The DTC model leverages causal learning to capture the interplay between static long-term preferences, dynamic short-term preferences, and next-item choices, enhancing predictive accuracy in session-aware recommender systems.

**Advantages**: By focusing on causal rather than associative relationships, the DTC model mitigates the risks of bias inherent in traditional methods, leading to more robust predictions.

**Limitations**: Although our assumptions about self-supervised learning are based on statistical findings from real-world data, these assumptions may not universally enhance model performance across all domains and datasets. The model design may not align with every scenario. Future work should consider tailoring the assumptions and model design to the specific characteristics of the dataset being used. This could involve adapting the self-supervised learning approach based on the domain’s unique features and user behavior patterns.

Our research primarily focused on the causal relationship between dynamic short-term preferences, static long-term preferences, and next-item recommendations. Thus, modeling user preferences was not the central aspect of this study. We employed basic methods for modeling dynamic short-term preferences and static long-term preferences, such as ignoring the sequential order and time-dependent features of interactions and using simple user embeddings for capturing static long-term preferences. These simplistic approaches are evidently inadequate and might limit the model’s performance. Future studies should explore more sophisticated user preference modeling techniques. Advanced methods that capture the sequential order and temporal characteristics of interactions, as well as enriched user embedding techniques, could significantly enhance the effectiveness of user preference models. Additionally, investigating causal relationships tailored to different user preference modeling approaches could further improve recommendation accuracy.

The DTC-SBRS model’s reliance on sufficient counterfactual data may limit its applicability in scenarios with sparse data or limited user interactions. Exploring advanced data augmentation techniques, leveraging transfer learning, or employing generative models to synthesize counterfactual data could mitigate challenges posed by data sparsity and enhance model robustness.

## 6. Conclusions

In summary, this paper offers a promising approach to enhancing session-aware recommendation systems through a causal lens. We have introduced a framework and model which, while still at an early stage, shows potential in addressing biases and improving predictions by considering long-term user preferences. Our empirical results demonstrate the DTC model’s superior performance in session-aware recommendation scenarios. The model effectively captures causalities between dynamic short-term, static long-term user preferences, and next-item choice, leading to more accurate and relevant recommendations. The demonstrated improvement in recommendation quality indicates the potential for significant impact when implemented in industrial applications, such as e-commerce platforms, music streaming services, and content recommendation systems. The DTC model’s robust performance on diverse datasets suggests its versatility across different domains.

Looking ahead, further work is needed to fully validate and refine our methods in diverse real-world scenarios. We hope to expand our research to include more varied data and contexts to test the generalizability and effectiveness of our approach. Additionally, we aim to explore the integration of our model with other machine learning techniques to unlock even richer, more personalized user experiences. Our pursuit for a deeper, causal understanding of user preferences in recommendation systems is just beginning, and we invite the research community to join us in this endeavor.

## Figures and Tables

**Table 1:** The detailed statistical information of the two datasets used in the experiments.

	Last.fm	Delicious
#sessions	5915	45,772
#interactions	38,367	249,919
#users	1101	1752
#items	711	5047
#interactions per user	34.85	142.65
#interactions per session	6.49	5.46
#sessions per user	5.37	26.13

**Table 2:** The evaluation results of all compared methods on two datasets. The metrics used for evaluation are Recall@5 (R@5), Recall@20 (R@20), NDCG@5 (N@5), NDCG@20 (N@20), MRR@5 (M@5), and MRR@20 (M@20). We employ 5-fold cross-validation and report the average values for each metric. Higher values indicate better performance for the evaluation metrics. The best performing method is indicated by bold numbers, while the second-best performing method is indicated by underlined numbers. An asterisk (*) denotes a significant improvement at a significance level of p<0.05.

	Last.fm						Delicious					
	R@5	N@5	M@5	R@20	N@20	M@20	R@5	N@5	M@5	R@20	N@20	M@20
SKNN	0.235	0.116	0.077	0.536	0.202	0.107	0.111	0.055	0.037	0.293	0.107	0.055
GRU4Rec	0.331	0.238	0.207	0.542	0.298	0.228	0.234	0.172	0.151	0.390	0.216	0.167
STAMP	0.269	0.190	0.164	0.500	0.256	0.187	0.150	0.104	0.089	0.294	0.105	0.103
CSRM	0.342	0.250	0.220	0.562	0.312	0.241	0.197	0.144	0.126	0.346	0.186	0.141
SR-GNN	0.265	0.186	0.160	0.477	0.246	0.181	0.205	0.149	0.130	0.354	0.191	0.145
HGRU	0.340	0.242	0.209	0.576	0.309	0.233	0.218	0.160	0.141	0.377	0.205	0.156
II-RNN	0.359	0.259	0.223	0.586	0.323	0.248	0.257	0.189	0.166	0.424	0.236	0.183
SASRec	0.346	0.206	0.141	0.651	0.201	0.178	0.193	0.130	0.071	0.385	0.184	0.093
BERT4Rec	0.304	0.182	0.142	0.622	0.273	0.174	0.207	0.101	0.084	0.369	0.186	0.100
INSERT	0.364	0.258	**0.224**	0.589	0.323	0.246	0.264	0.196	0.174	0.436	0.245	0.191
COCO	0.504	0.289	0.205	0.793	0.374	0.238	0.359	0.215	0.163	0.520	0.263	0.180
DTC	0.519 *	0.298 *	**0.224**	0.814 *	0.385 *	0.256 *	0.385 *	0.229 *	0.177 *	0.564 *	0.282 *	0.196 *

**Table 3:** Recommendation performance of DTC and its simplified variants on Delicious and Lastfm. The best performing method is indicated by bold numbers.

Dataset	Variant	R@5	N@5	R@20	N@20
Last.fm	DTC w/o CF	0.325	0.201	0.643	0.292
	DTC w/o DT	0.504	0.289	0.793	0.374
	DTC w/o SSL	0.510	0.295	0.809	0.382
	DTC	**0.519**	**0.298**	**0.814**	**0.385**
Delicious	DTC w/o CF	0.214	0.137	0.397	0.190
	DTC w/o DT	0.359	0.215	0.520	0.263
	DTC w/o SSL	0.333	0.206	0.523	0.262
	DTC	**0.385**	**0.229**	**0.564**	**0.282**

## Data Availability

No new data were created or analyzed in this study. Data sharing is not applicable to this article.
